# Modeling CSF circulation and the glymphatic system during infusion using subject specific intracranial pressures and brain geometries

**DOI:** 10.1186/s12987-024-00582-0

**Published:** 2024-10-15

**Authors:** Lars Willas Dreyer, Anders Eklund, Marie E. Rognes, Jan Malm, Sara Qvarlander, Karen-Helene Støverud, Kent-Andre Mardal, Vegard Vinje

**Affiliations:** 1 Department of Scientific Computing and Numerical Analysis, Simula Research Laboratory, Oslo, Norway; 2 Department of Diagnostics and Intervention, Biomedical engineering and radiation physics, Umeå University, Umeå, Sweden; 3 Department of Mathematics, University of Oslo, Oslo, Norway; 4 Department of Health Research, SINTEF Digital, Trondheim, Norway; 5 Expert Analytics AS, Oslo, Norway; 6 Department of Clinical Sciences, Umeå University, Umeå, Sweden; 7 KG Jebsen Center for Brain Fluid Research, Oslo, Norway; 8 BI Norwegian Business School, Oslo, Norway

**Keywords:** Infusion test, CSF circulation, Glymphatic pathway, CSF dynamics, Intracranial pressure, Paravascular flow

## Abstract

**Background:**

Infusion testing is an established method for assessing CSF resistance in patients with idiopathic normal pressure hydrocephalus (iNPH). To what extent the increased resistance is related to the glymphatic system is an open question. Here we introduce a computational model that includes the glymphatic system and enables us to determine the importance of (1) brain geometry, (2) intracranial pressure, and (3) physiological parameters on the outcome of and response to an infusion test.

**Methods:**

We implemented a seven-compartment multiple network porous medium model with subject specific geometries from MR images using the finite element library FEniCS. The model consists of the arterial, capillary and venous blood vessels, their corresponding perivascular spaces, and the extracellular space (ECS). Both subject specific brain geometries and subject specific infusion tests were used in the modeling of both healthy adults and iNPH patients. Furthermore, we performed a systematic study of the effect of variations in model parameters.

**Results:**

Both the iNPH group and the control group reached a similar steady state solution when subject specific geometries under identical boundary conditions was used in simulation. The difference in terms of average fluid pressure and velocity between the iNPH and control groups, was found to be less than 6% during all stages of infusion in all compartments. With subject specific boundary conditions, the largest computed difference was a 75% greater fluid speed in the arterial perivascular space (PVS) in the iNPH group compared to the control group. Changes to material parameters changed fluid speeds by several orders of magnitude in some scenarios. A considerable amount of the CSF pass through the glymphatic pathway in our models during infusion, i.e., 28% and 38% in the healthy and iNPH patients, respectively.

**Conclusions:**

Using computational models, we have found the relative importance of subject specific geometries to be less important than individual differences in resistance as measured with infusion tests and model parameters such as permeability, in determining the computed pressure and flow during infusion. Model parameters are uncertain, but certain variations have large impact on the simulation results. The computations resulted in a considerable amount of the infused volume passing through the brain either through the perivascular spaces or the extracellular space.

## Introduction

Idiopathic Normal Pressure Hydrocephalus (iNPH) is a partially reversible neurological disorder characterized by enlarged ventricles. Typical symptoms are gait disturbance, urinary incontinence, and cognitive decline which may improve after shunt treatment [1]. Infusion tests have been shown to be an effective procedure for predicting if a patient is going to respond well to treatment [2, 3]. An infusion test measures the outflow resistance, $R_{out}$, of the cerebrospinal fluid system as a whole. This resistance is in general significantly larger in iNPH patients than in healthy individuals [2, 4]. Additional quantities like compliance, time required to reach pressure equilibrium, and craniospinal pressure volume index (PVI) are also common indicators [5, 6].

In 2012 [7] the glymphatic pathway for cerebrospinal fluid flow through the murine brain was identified and suggested to be important for clearance of metabolic waste. Accumulation of metabolic waste is common in dementia [8, 9] and is suggested to be caused by a malfunctioning glymphatic pathway. The glymphatic system and its pathways have been detailed in mice, both on the micro-scale with 2-photon imaging and on the macro-scale with magnetic resonance imaging (MRI), see [10] for an overview. Here, evidence suggest that bulk CSF flow occurs in pial PVS [11–13] and these flow velocities are around 20 $\mu$m/s. Bulk flow of interstitial fluid has been estimated in the range from 10 nm/s [14–16] to 1.7 $\mu$m/s [17, 18]. In humans, less is known about the glymphatic pathways, although, in particular contrast-enhanced MRI provide a detailed macroscopic perspective on how the enhanced transport provided by the PVS is three times greater than that of extra-cellular diffusion [19]. Here, iNPH patients are particularly interesting as the relation between outflow resistance and pressure during infusion tests is well characterized in this patient group. Furthermore, the CSF dynamics of the brain is significantly altered in patients suffering from iNPH. Pulsatile flow of CSF in the aqueduct of Sylvus is larger in iNPH patients than in healthy individuals. Furthermore, the net flow is potentially retrograde rather than antegrade [20, 21]. Finally, tracer intrathecally injected into the brain has a significantly delayed clearance rate in iNPH patients [21–23]. If untreated, iNPH might lead to irreversible damage to brain tissue, and hence early detection and intervention is crucial [24]. Infusion testing is both a reliable and frequently used method for selecting patients for surgery [2, 25, 26].

There exist several computational modeling studies [27–30] of hydrocephalus and iNPH which predate the glymphatic system by Iliff et al.[7]. These studies focus on the interstitial space, with the intracranial pressure given as boundary conditions and do not include perivascular pathways. To consider the glymphatic pathway, Vinje et al. [31] constructed a 0D multi-compartmental model investigating how fluid flow patterns change in the brain during infusion tests. Furthermore, Guo et al. [32] utilized a model similar to ours to study both the glymphatic pathway and subject specific geometries to model cerebral CSF dynamics, but they did not consider infusion tests. Finally, we mention that the mathematical theory for such systems has recently been studied in several papers, see Lee et al. [33] for an overview. However, so far, the relative importance between (1) subject specific geometries (2) intracranial pressure and (3) physiological parameters has not been assessed by computational models.

In this study we explored the differences between iNPH patients and healthy controls during an infusion test in terms of fluid pressure, speed and flow in the CSF and Interstitial Fluid (ISF). In 47 subject specific geometries, we studied fluid pressure in the perivascular spaces (PVS) and the extracellular space (ECS) of the brain, as well as ECS water transport to blood networks and blood perfusion. First, we tested whether the difference in geometry alone was sufficient to obtain differences in intracranial pressure (ICP) between the two groups during infusion. Second, we investigated if subject specific boundary conditions, in terms of subarachnoid CSF pressure and arterial inflow, are important for our model. Finally, we investigated if changes to brain physiology and the glymphatic pathway may play a role in the iNPH response to infusion. These changes included variations to permeability in the ECS and PVS, and variations in fluid flow pathways between the PVS and ECS.

## Methods

**Table 1:** Characteristic data and range for the iNPH and control cohorts   [34]

Parameters	Healthy ($N=35$)	iNPH ($N=16$)
Sex (F/M)	19/16	7/9
Age (years)	71 ± 5 (64-81)	73±5 (64-82)
Mini-mental state examination (MMSE)	29 ± 1 (28-30)	27 ± 3 (21-30)
Ventricular volume (ml)	43 ± 20 (16-99)	181 ± 60 (95-310)
Evans index	0.29 ± 0.03 (0.23-0.36)	0.39 ± 0.04 (0.33-0.49)
Aqueduct cross-sectional area (cm$^2$)	0.10 ± 0.02 (0.09-0.16)	0.23 ± 0.17 (0.09-0.77)
Heart rate (bpm)	64±9 (47-85)	72±11 (52-87)
Brain volume (l)	1.04 ± 0.10	1.03 ± 0.08
Pial surface area (dm$^2$)	20.5 ± 1.75	20.4 ± 1.71
Ventricular surface area (dm$^2$)	1.89 ± 0.43	3.30 ± 0.47
Outflow resistance $R_{out}$ (mmHg/(ml/min))	10.03 ± 4.71	18.06 ± 7.84
Arterial inflow $B_{in}$ (ml/min)	712.5 ± 172.4	653.4 ± 172.2
Reference pressure $p_{ref}$ (mmHg)	8.92 ± 2.48	9.26 ± 3.08

Table shows average value with ± one standard deviation. The reference pressure $p_{ref}$ is the steady state pressure at infinite compliance [35]

### Subject data and mesh generation

From previous studies [36, 37] we obtained T1-weighted MRI images (turbo field echo (T1W-TFE) sequence) and pressure measures of 47 subjects (33 healthy and 14 iNPH patients), see Table 1. The MR images were taken with a 3T Philips Achieva scanner (Philips Healthcare, Best, the Netherlands) with resolution $1.0 \times 1.0 \times 1.0$ mm, which was interpolated to get an image with resolution $0.3 \times 0.3 \times 1.0$ mm. Both the iNPH patients and the controls underwent an infusion test where mock CSF is injected into the lumbar canal. As the CSF volume increases, the ICP rises and parameters such as $R_{out}$ are measured [6, 38].

**Fig. 1:**
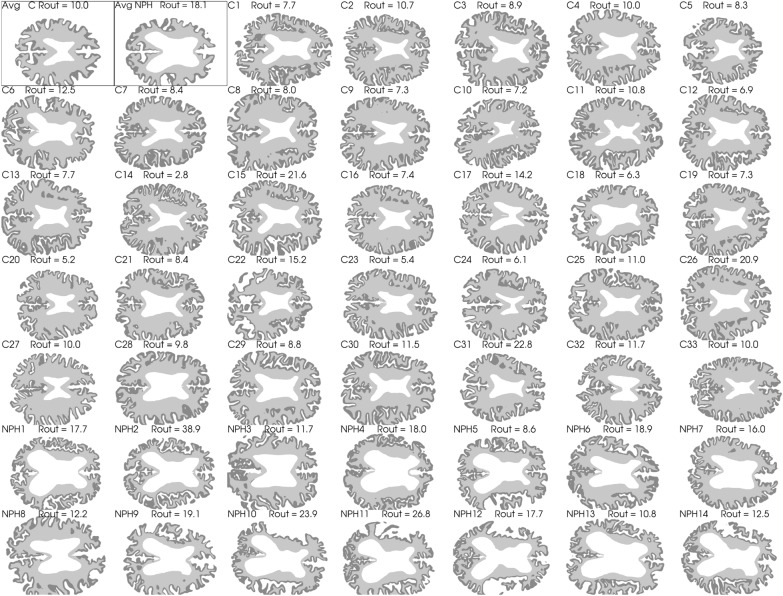
Axial slices of each subject as well as the average geometry marked as Avg C and Avg NPH for the control and iNPH groups respectively. The segmented gray matter is shown in dark gray, and the white matter is shown as a lighter shade of gray. The white space in the middle show the lateral ventricles. The two average geometries are shown first in the top left corner and are highlighted with a thin black frame

We considered the two groups both on subject specific level and group level. At the group level we created average images of the control and iNPH group, respectively. The images were preprocessed using Statistical Parametric Mapping software (SPM12; Wellcome Department of Cognitive Neurology, University College London, London, United Kingdom). First, the T1-images were segmented into gray matter, white matter, and CSF. Then, we used DARTEL [39] for image registration into group-specific templates (averages) of the control and iNPH group, respectively. The normalized images were finally aligned into MNI space and smoothed using a 1.0 mm full width at half maximum Gaussian filter.

We performed a Freesurfer [40] segmentation on each of the subject specific images and on the average images. Based on the Freesurfer segmentation, we generated computational meshes which we used in the numerical simulations. Only the segmentations for the brain stem, and the gray and white matter in the cerebrum and cerebellum was used in the mesh generation process, and the meshes were generated using SVMTK [41, 42], an axial slice of each brain geometry is shown in Fig. 1. One 3D mesh was created for each patient, consisting of three subregions given by the segmented white matter, gray matter and brain stem.

**Fig. 2:**
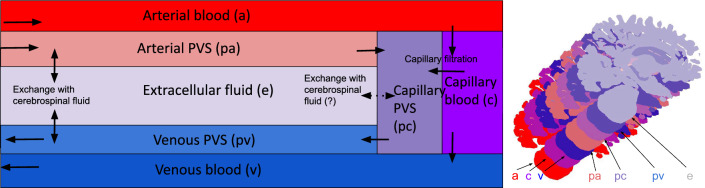
Conceptual sketch showing each compartment and their connections. Blood enters the brain through the arterial compartment and flows into the capillaries. From there, most of the blood flows further to the venous compartment and then out from the brain parenchyma, but a small fraction enters the perivascular space by means of capillary filtration. This is one of the two entryways for fluid into the PVS, the other being inflow through the arterial PVS. From there, the CSF can either keep flowing through the perivascular spaces, going first to the capillary PVS before travelling further to the venous PVS from which it leaves the parenchyma through pial sleeves alongside the cerebral veins. Alternatively, the CSF can flow through the ECS and to the venous PVS. In our base model, the possible fluid exchange between the ECS and capillary PVS was assumed to not be present, and is therefore marked with a dotted arrow. We remark that the focus here is on the compartments of the parenchyma and that the various exit routes are lumped together through one ordinary differential equation enforced uniformly at the brain surface

### Governing equations

We modelled the brain as a porous medium using a modified version of the MPET framework [29], where elastic deformations are ignored. Our model consists of seven compartments, namely arterial (a), capillary (c), and venous (v) blood compartments, their corresponding perivascular compartments (pa, pc, pv), and the extracellular space (e). Deformations of the brain parenchyma during an infusion were assumed to be negligible, yielding a simplified set of equations [43] given by

1 $$\begin{aligned} C(p_i{(\textbf{x},t)})\frac{ \partial p_i}{\partial t}{(\textbf{x},t)} - \nabla \cdot \frac{\kappa _i}{\mu _i}\nabla p_i{(\textbf{x},t)} = {\sum _{\underset{j\in \mathcal {I}}{j\ne i}} {F_{i,j}}(\textbf{x},t)}. \end{aligned}$$

Here, $p_i$ is the pressure in compartment *i*, while $C(p_i)$, $\kappa _i$ and $\mu _i$ denotes the compartmental compliance, permeability and viscosity, respectively. The arguments taken by $p_i$ and $F_{i,j}$, denoted by $\textbf{x},t$ represent the spatial coordinate vector (*x*, *y*, *z*) and time respectively. The sum of the ${F_{i,j}}(\textbf{x},t)$ terms denotes the total fluid transfer rate between compartment *i* and its connected compartments and was modelled following  [29, 44] by:

2 $$\begin{aligned} {\sum _{\underset{j\in \mathcal {I}}{j\ne i}} {F_{i,j}}(\textbf{x},t)} = \sum _{\underset{j\in \mathcal {I}}{j\ne i}}\omega _{i,j}(p_j{(\textbf{x},t)} - p_i{(\textbf{x},t))}, \end{aligned}$$

with $\mathcal {I}$ being the set of compartments, and $\omega _{i,j}$ being the transfer coefficient between compartment *i* and *j*. The pressure fields computed in the governing equations can be used to find the superficial velocity (porosity scaled) in each compartment, $\textbf{v}_i{(\textbf{x},t)}$, defined by

3 $$\begin{aligned} \textbf{v}_i{(\textbf{x},t)}^n = -\frac{\kappa _i}{\mu _i \phi _i} \nabla p_i^n{(\textbf{x},t)}, \end{aligned}$$

where $\phi _i$ is the compartmental porosity. In all figures and numbers where fluid speed is mentioned, we are referring to the volume averaged superficial speed (magnitude of the superficial velocity vector). The model is illustrated graphically in Fig. 2.

### Parameters

In the governing equations, we use values from the literature to determine the permeabilities and porosities of each compartment as well as the intercompartmental transfer coefficient. Following [29], we set $\mu _\text {blood} = 3\mu _\text {CSF/ISF}$, and the viscosity of CSF and ISF was set to the viscosity of water at 37 degrees centigrade. Finally, we assumed negligible deformations, and a low compliance $C_i$ for all compartments. Larger vessels, namely arteries and veins were given larger compliance of $C_\text {a} = C_\text {v} = 10^{-4}$ Pa$^{-1}$, while the remaining five compartments have a compliance of $C_i = 10^{-8}$ Pa$^{-1}$.

Estimates for the flow resistance between different cerebral compartments are based on [31], whose model defined the intercompartmental transfer by

4 $$F_{{i,j}} ^{{0D}} (t) = \frac{1}{{R_{{i,j}} }}\left( {p_{j}^{{0D}} \left( t \right) - p_{i}^{{0D}} (t)} \right),{\text{ }}$$

with ${p_i^{0D}}{(t)}$ being the pressure in each 0D-compartment. Taking the volume average integral of Equation (2), we can relate ${\omega _{i,j}}$ to the zero dimensional resistance ${R_{i,j}}$ by

5 $$\begin{aligned} \omega _{i,j} = \frac{1}{V_\Omega R_{i,j}}. \end{aligned}$$

Here, $V_\Omega$ is the volume of the computational domain. The transfer coefficients for flow between the PVS and the ECS, and the coefficients for flow between the PVS and capillary compartment were computed using the estimates of  [31] for the one dimensional resistance $R_{i,j}$.

We assumed the pressure drop between the different blood networks to be constant in time and equal between patients. This allowed us to determine the inter-compartmental transfer coefficients $\omega _{i,j}$ by equating Equation (2) to the arterial inflow rate $B_\text {in}$. Relating the transfer coefficient between blood vessels to the expected pressure drop $\Delta {\bar{p}_{i,j}}{(t)} = \overline{(p_i - p_j)}{(t)}$, we get

6 $$\begin{aligned}&\omega _\text {a,c} \Delta {\bar{p}_\text {a,c}} V_\Omega = B_\text {in}, \end{aligned}$$

7 $$\begin{aligned}&\omega _\text {c,v} \Delta {\bar{p}_\text {c,v}} V_\Omega = B_\text {in}. \end{aligned}$$

Here, the overbar denotes the volume average operator. We assumed the pressure drop to be constant in time before infusion, with a magnitude of 60 mmHg from the arterial to the capillary compartment  [45, 46], and 10 mmHg from the capillaries to the venous compartment  [46, 47]. The transfer coefficients depend on brain volume and arterial inflow, and hence we have listed the coefficients for a brain with $V_\Omega = 1000$ ml and $B_{in} = 700$ ml/min in Table 2. These values were chosen to be similar to the data we used in our simulation, but both volume and blood flow varied between subjects and cohorts.

The resistance to fluid flow in each of our compartments from [31] can be converted to permeabilites for a three-dimensional compartment [31, 43]. If we let $\Delta p$ denote the steady state pressure change over a porous channel of length *L* and cross sectional area *A*, then the volume flux of a fluid with steady volumetric flux ${\textbf{q}(\textbf{x})}$ through this channel is given by

8 $$\begin{aligned} Q = \int _A \textbf{q}{(\textbf{x})} \cdot \textbf{n} dS = \frac{\kappa \Delta p A}{L\mu }. \end{aligned}$$

Here, $\textbf{n}$ denotes the surface unit normal vector. Following [31], we let $R_i = \Delta p_i/Q_i$ denote the flow resistance in compartment *i*. We assumed homogeneity of the brain tissue, and that *L* and *A* are equal in all compartments. Using Equation (8) with the definition of $R_i$, the permeability and viscosity are related by

9 $$\begin{aligned} \frac{R_i \kappa _i}{\mu _i} = \frac{L}{A} = \text {constant}. \end{aligned}$$

The permeability of the ECS, $\kappa _{e}$, was estimated to be in the range spanning from 10 nm$^2$ by [14] to 4500 nm$^2$ [48, 49]. The authors do note that other estimates of the permeability reaches values in the order of 1000 nm$^2$ to 4000 nm$^2$ [50–53], but none of these latter references distinguish between perivascular and extracellular spaces. Hence, letting the extracellular permeability $\kappa _\text {e}$ be 20 nm$^2$, $\mu _\text {ISF} = \mu _\text {CSF} = 0.75$ mPa$\cdot$s all together yields $L/A = 1.21 \cdot 10^{-4}$ m$^{-1}$ according to Eq. (9).

In their computation of pericapillary resistance, Vinje et al. [31] considered two scenarios. One high resistance scenario where the capillary PVS was assumed to be 100 nm wide, based on the measurements of Bedussi et al. [54]. These measurements were, however, conducted using fixation, which has been shown to shrink the PVS [11]. Furthermore, Pizzo et al. [55] were able to image a part of the cerebral microvasculature of mice. Their image apparently show a PVS width of more than 1 $\mu$m. Hence, we have in our model assumed the extended capillary gaps scenario from Vinje et al. [31] to be more representative of the actual physiology. We have listed the final values for each compartmental permeability in Table 2.

**Table 2:** Compartmental material parameters used in our base model

Parameter	Value	Units	Source
$\omega _\text {a,c}$	$1.45 \cdot 10^{-6}$	Pa$^{-1}$s$^{-1}$	[46]
$\omega _\text {c,v}$	$8.75 \cdot 10^{-6}$	Pa$^{-1}$s$^{-1}$	[47]
$\omega _\text {c,pc}$	$8.48 \cdot 10^{-10}$	Pa$^{-1}$s$^{-1}$	[31]
$\omega _\text {pa,e}$	$1.86 \cdot 10^{-7}$	Pa$^{-1}$s$^{-1}$	[31]
$\omega _\text {pv,e}$	$1.65 \cdot 10^{-7}$	Pa$^{-1}$s$^{-1}$	[31]
$\omega _\text {pa,pc}$	$10^{-6}$	Pa$^{-1}$s$^{-1}$	Estimated
$\omega _\text {pc,pv}$	$10^{-6}$	Pa$^{-1}$s$^{-1}$	Estimated
$\omega _\text {pc,e}$	$10^{-10}$	Pa$^{-1}$s$^{-1}$	Estimated
$\kappa _\text {a}$	$3.63 \times {10^{4}}$	nm$^2$	[31, 56]
$\kappa _\text {c}$	$1.44\times {10^{3}}$	nm$^2$	[57]
$\kappa _\text {v}$	$1.13 \times {10^{6}}$	nm$^2$	[31, 56]
$\kappa _\text {e}$	20	nm$^2$	[14]
$\kappa _\text {pa}$	30	nm$^2$	[31, 56]
$\kappa _\text {pc}$	$1.44 \times {10^{3}}$	nm$^2$	[57]
$\kappa _\text {pv}$	$1.95 \times {10^{4}}$	nm$^2$	[31, 56]
$\phi _\text {a}$	$1.09 \cdot 10^{-2}$	–	[58, 59]
$\phi _\text {c}$	$2.31 \cdot 10^{-3}$	–	[58]
$\phi _\text {v}$	$1.98 \cdot 10^{-2}$	–	[58]
$\phi _\text {pa}$	$1.52 \cdot 10^{-2}$	–	[11]
$\phi _\text {pc}$	$2.31 \cdot 10^{-3}$	–	[55]
$\phi _\text {pv}$	$2.77 \cdot 10^{-2}$	–	[11]
$\phi _\text {e}$	$1.40\cdot 10^{-1}$	–	[60]

The porosities are dimensionless and are therefore marked with -

The cerebral blood volume fraction was set at 3.3% of the total brain volume, [58, 59, 61] of which a third of the total cerebral blood volume (CBV) is arterial blood [58, 59]. We assumed a capillary blood fraction of 10% of total CBV, with the remaining 57% being venous blood [58, 59]. These volume fractions were used directly to determine the porosities for the vascular compartments.

The perivascular porosities were computed as the relative size fraction between the PVS and its corresponding vasculature. We have for the arterial and venous perivascular spaces assumed the porosity to be 1.4 times that of the vasculature, as observed to be the area ratio on the pial surface in mice  [11]. While the penetrating PVS might be smaller than the surface PVS, this proportionality gives a tangible upper bound on the porosities and hence a lower bound on the computed fluid velocities. For the capillary PVS, we used an ex-vivo image of the perivascular space of a cerebral microvessel [55]. This image suggest a porosity proportionality of 1 between the pericapillary space and the capillaries. Finally, we have used an ECS porosity $\phi _{e} = 0.14$, based on observations made of the murine brain [60]. All porosities are listed in Table 2.

The subarachnoid cerebrospinal fluid pressure, $p_\text {CSF}$ was computed using the model of [31]:

10 $$\begin{aligned} C(p_\text {CSF}{(t)})\frac{\partial p_\text {CSF}}{\partial t}{(t)}&= Q_\text {prod} + Q_\text {inf}{(t)} + Q_\text {pvs}{(t)} \nonumber \\ {}&- \frac{1}{R_\text {DS}}(p_\text {CSF}{(t)} - p_\text {DS}) \\ {}&- \frac{1}{R_\text {crib}}(p_\text {CSF}{(t)} - p_\text {crib}). \nonumber \end{aligned}$$

Here $p_\text {DS} = 8.4$ mmHg denotes the pressure at the dural sinus and $R_\text {DS} = 10.81$ mmHg/(mL/min) the corresponding resistance, while $p_\text {crib} = 0$ (atmospheric pressure) and $R_\text {crib} = 67$ mmHg/(mL/min) is the cribriform plate pressure and resistance [31]. Furthermore, $Q_\text {inf}, Q_\text {prod}$ and $Q_\text {pvs}$ denote the CSF in- and outflow from infusion, production in choroid plexus and outflow to the cerebral PVS respectively. The function $C(p_{CSF}{(t)}$ is the subarachnoid compliance, and was modelled following [31]:

11 $$\begin{aligned} C(p_\text {CSF}{(t)}) = {\left\{ \begin{array}{ll}\frac{1}{E(p_\text {lim} - p_\text {ref})}, &{} p_\text {CSF}{(t)} < p_\text {lim}, \\ \frac{1}{E(p_\text {CSF}{(t)}-p_\text {ref})}, &{} p_\text {CSF}{(t)} > p_\text {lim}.\end{array}\right. } \end{aligned}$$

Here, $E=0.2$ ml$^{-1}$ is the elastance of the system, $p_0 = 13$ mmHg is a lower threshold pressure and $p_\text {ref}$ = 9 mmHg [31] in the base model. In cases where $p_0$ or *p* were smaller than $p_\text {ref}$, the reference pressure was set to be 1 mmHg less than $p_0$ or *p*. We remark that $Q_\text {pvs}{(t)}$ was not included in [31], but was included here to ensure mass conservation.

The infusion test measurements from [34] were used to tune the boundary conditions of our computational model by fitting the best parameters of a lumped ordinary differential equation. In detail, we assumed the outflow resistances of the dural sinus and the cribriform plate, $R_\text {DS}$ and $R_\text {crib}$, to depend linearly on a real number $\alpha$, i.e., $R_\text {DS}(\alpha ) = R_\text {DS}^\text {const} \cdot \alpha$ and $R_\text {crib} = R_\text {crib}^\text {const} \cdot \alpha$. Here, the const-superscript indicates that it is a constant with a value equal to those used by [31]. We computed the pressure curves predicted by Equation (10) for an infusion test with $Q_\text {inf}{(t)} = 1.5$ ml/min for different values of $\alpha$. Following  [31], only $Q_\text {prod}$ and $Q_\text {inf}{(t)}$ were included in these computations. Then, we computed the total $R_\text {out}$ attained for each value of $\alpha$. Using linear regression, we were able to then relate $R_\text {out}$ to $\alpha$ and found the following relation to be the best fit

12 $$\begin{aligned} R_\text {out} = 11.3\alpha - 1.3. \end{aligned}$$

The mean squared error of the fit was $1.8\cdot 10^{-4}$ mmHg/(ml/min). Finally, the compliance $C(p_\text {CSF}{(t)})$ was tuned to depend on subject specific reference pressures $p_\text {ref}$, modelling interpersonal variance in infusion response time.

### Simulation setup

**Fig. 3:**
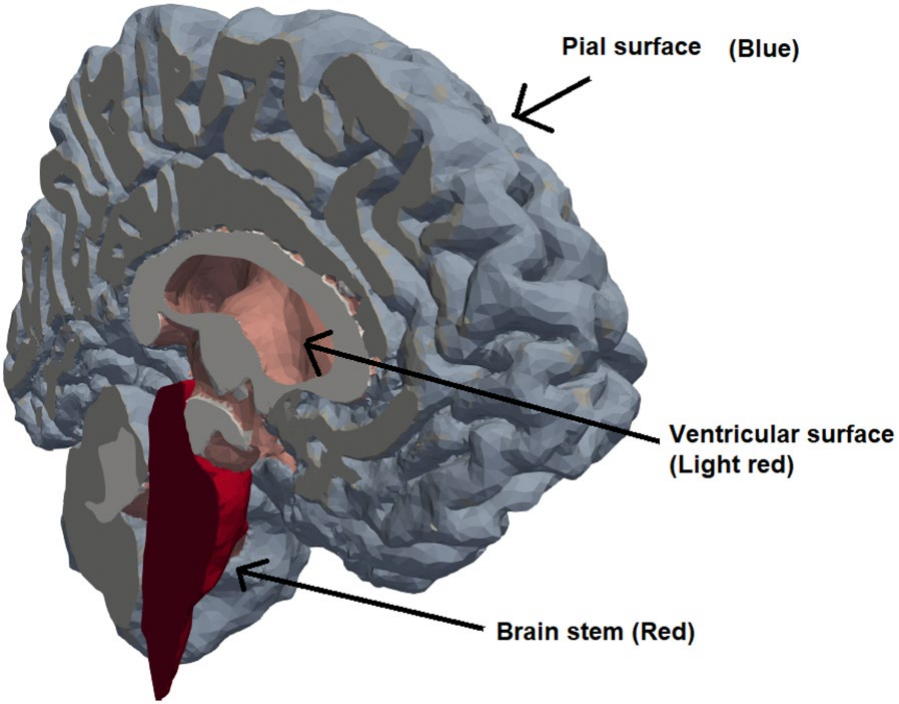
The surfaces of the computational geometry. The in- and outflow in the vascular and perivascular spaces went through the pial surface (blue), while fluid secretion from capillaries to choroid plexi was enforced through the ventricular walls

Our domain was partitioned in three subdomains, namely gray matter, white matter and the brain stem. On the latter, homogeneous Neumann boundary conditions were used, ensuring no flow through the boundary of this part of the domain. The extracellular and pericapillary compartments were assumed disconnected from the subarachnoid CSF due to barriers such as pia and glia limitants, and had a homogenous Neumann condition on the entirety of the boundary surface. No compartment had fluid flow through more than one surface, and hence all had a no-flow condition on either the pial or ventricular surface. The boundary conditions which are not homogenous Neumann are listed in Table 3, and the surfaces where these boundary conditions were applied are shown in Fig. 3. We enforced two sets of boundary conditions, generic and subject specific. These two cases differ in which values for arterial inflow $B_\text {in}$, outflow resistance $R_\text {out}$ and reference pressure $p_\text {ref}$ were used. In the generic case, the average value in the control group was used for each of the aforementioned values. In the subject specific case, these parameters were set based on individual measurements.

The arterial and venous boundary conditions enforce a constant flow rate through the brain parenchyma, entering over the entire pial surface and leaving through veins penetrating the cortical surface. The boundary condition on the capillaries transported a fixed amount of fluid through the ventricular surface, modelling the fluid secretion by choroid plexi for CSF production. The perivascular boundary condition ensured that CSF flow through the PVS is driven by a pressure gradient between the PVS and SAS. On the venous PVS, the average of the Dural Sinus pressure and the subarachnoid CSF pressure is used to model the general increase in CSF pressure across the entire CNS, while the CSF still leaves the parenchyma alongside the cerebral veins. The parameters $\beta _1, \beta _2$ and $\beta _3$ are numerical coefficients with units m/(s$\cdot$ mmHg) which determine the velocity to pressure difference relation through the surface boundary. These parameters were set to ensure an expected pressure curve development during infusion and relaxation period post-infusion.

**Table 3:** Table of each in- and outflow boundary conditions enforced on each compartment in the model

Compartment	Boundary condition	Surface	Notes
Arterial	${\frac{\kappa _\text {a}}{\mu _\text {blood}}}\nabla p_\text {a}{(\textbf{x},t)}\cdot \hat{\mathbf{n}} = Q_\text {in}$	Pial	$Q_\text {in} = B_\text {in}/A_\text {pial}$.
Capillary	${\frac{\kappa _\text {c}}{\mu _\text {blood}}}\nabla p_\text {c}{(\textbf{x},t)} \cdot \hat{\mathbf{n}} = -Q_\text {prod}$	Ventricular	$Q_\text {prod} = 0.33$ ml/min.
Venous	${\frac{\kappa _\text {v}}{\mu _\text {blood}}} \nabla p_\text {v}{(\textbf{x},t)} \cdot \hat{\mathbf{n}} = \beta _1 \left( \dfrac{p_\text {DS} + p_\text {CSF}{(t)}}{2} - p_\text {v}{(\textbf{x},t)}\right)$	Pial	$p_\text {DS} = 8.4$ mmHg, $\beta _1 = {1.33\cdot 10^{-1}}$ m/(s$\cdot$mmHg).
Arterial PVS	${\frac{\kappa _\text {pa}}{\mu _\text {CSF}}}\nabla p_\text {pa}{(\textbf{x},t)} \cdot \hat{\mathbf{n}}= \beta _2(p_\text {CSF}{(t)} - p_\text {pa}{(\textbf{x},t))}$	Pial	$\beta _2 = {1.33\cdot 10^{-1}}$ m/(s$\cdot$mmHg).
Venous PVS	${\frac{\kappa _\text {pv}}{\mu _\text {CSF}}}\nabla p_\text {pv}{(\textbf{x},t)} \cdot \hat{\mathbf{n}} = \beta _3(\dfrac{p_\text {CSF}{(t)} + p_\text {DS}}{2} - p_\text {pv}{(\textbf{x},t)})$	Pial	$\beta _3 = {1.33\cdot 10^{-5}}$ m/(s$\cdot$mmHg).

Here, $A_\text {pial}$ is the computed pial surface area of the mesh and $p_\text {CSF}(t)$ is the pressure in the subarachnoid space, and is defined in Equation (10)

Each simulation ran for $T=70$ minutes. The simulation is run in three stages, with the first 16 min and 40 s being run without infusion to let the system reach steady state before turning on infusion. After this initial period, at $t=0$ min, infusion starts at a constant rate of $Q_\text {inf} = 1.5$ ml/min. The infusion test lasts for 33 min and 20 s. Afterwards, when infusion stops the simulation runs for a period of 20 min to check for a return to the original steady state. The stage time length was set based on computed convergence time in terms of fluid velocity in a single subject.

The meshing software SVMTK uses a resolution parameter (RP) to determine an upper bound on cell size. Using numerical convergence testing, an RP of 32 (typically around 1.1M cells) was found to be sufficient for convergence with second order Lagrange polynomials as finite element basis functions, with a less than 2 % change in plateau pressure from a RP16 resolution (data not shown). The equations were discretized in time using a Backward-Euler scheme. A convergence test of temporal resolution was performed with time steps ranging from 180 s to 15 s. The simulation converged with all choices of time steps, and a time step of $\Delta t = 20\,s$ was sufficient to achieve convergence in terms of a negligible change in the computed pressure curves (compared to the 15 s time step, determined graphically). All details regarding discretisation, mesh and time resolution can be found in [43], chapter 6. The simulations were performed using the Legacy FEniCS solver for python. [62, 63]

We performed eight sets of simulation experiments, listed in Table 4. In the first three simulation sets, labelled as base model 1, 2 and 3, we explored the effect of geometric differences and differences in boundary conditions. Here, we used either subject specific or average geometries and either subject specific or generic boundary conditions. The difference in subject specific and generic boundary conditions lies in whether subject specific or the control group average value for $B_\text {in}$, $p_\text {ref}$ and $R_{out}$ was used in Equation (12), Equation (10), Equation (11) and Table 3. The generic boundary conditions used the average values for the control group for these parameters. The last set of simulations, labelled variation 1 to 5 investigated how changes to material parameters affected the response to infusion.

**Table 4:** Overview of the eight different sets of simulations that were performed in this study

Experiment	Geometry	BC	Other changes	# Simulations
Base model 1	Subject specific	Generic	None	47
Base model 2	Subject specific	Subject specific	None	47
Base model 3	Average	Subject specific	None	47
Variation 1	Average	Generic	Pericapillary resistance set to either (in mmHg/(ml/min)): $R_{pc} = 9.2\cdot 10^{-3}$ (Base), $R_{pc} = 3.32\cdot 10^{-4}$ (Pizzo) or $R_{pc} = 32.24$ (High).	3
Variation 2	Average	Generic	CSF outflow resistances changed to (in mmHg/(ml/min)): $R_{DS}= 10.81, R_{crib} = 67$ (Base), $R_{DS}= 21.62, R_{crib} = 67$ (High), $R_{DS}= 5.41, R_{crib}= 67$ (Low).	3
Variation 3	Average	Generic	Changes to capillary filtration: $\omega _{c,pc} = 8.48\cdot 10^{-10}$ Pa$^{-1}$s$^{-1}$ , no constant filtration (Base), or $\omega _{c,pc} = 0$ Pa$^{-1}$s$^{-1}$ , with constant filtration rate of 0.16 ml/min at ventricles (Alternate).	2
Variation 4	Average	Generic	Perivascular transfer coefficients changed to (units Pa$^{-1}$s$^{-1}$): $\omega _{pa,e}= 1.86\cdot 10^{-7}$, $\omega _{e,pv} = 1.65\cdot 10^{-7}$, $\omega _{pc,e}= 10^{-10}$ (Base), $\omega _{pc,e}= 5\cdot 10^{-7}$, the rest unchanged (Case 1), $\omega _{pa,e}= 1.86\cdot 10^{-6}$, $\omega _{e,pv} = 1.65\cdot to^{-6}$, $\omega _{pc,e}= 10^{-8}$ (Case 2), $\omega _{pa,e}= 10^{-10}$, $\omega _{e,pv}$ unchanged, $\omega _{pc,e}= 1.86\cdot 10^{-7}$ (Case 3), $\omega _{pa,e}$ halved, $\omega _{e,pv}$ halved, $\omega _{pc,e}$ unchanged (Case 4).	5
Variation 5	Average	Generic	Changes to extracellular permeability (Units nm$^2$): $\kappa _{e} = {20}$ (Base), $\kappa _{e} = {200}$ (High), $\kappa _{e} = {2000}$ (Very high).	3

In the model variation simulations, we implemented the changes to the material parameters on a single brain, the average control brain. Each model variation is listed in Table 4, and is explained briefly in the following paragraphs.

#### Variation 1: Pericapillary channel width variations

Due to their small size and their location, the width of capillary PVS is debated. In these simulations we assessed how changes to pericapillary permeability and porosity changed the flow patterns in the brain before, during and after infusion. Using equation 8 from [31], we found the pericapillary resistance predicted by the image of Pizzo et al. (2018) to be $R_{pc} = 3.32 \cdot 10^{-4}$ mmHg/(ml/min). Two additional possibilities for pericapillary resistances is discussed by Vinje et al. (2020), namely a high resistance scenario based on [54] which suggest a $R_{pc} = 32.24$ mmHg/(ml/min), and a low resistance scenario where the resistance is computed to be $R_{pc} = 9.2 \cdot 10^{-3}$ mmHg/(ml/min). In this model variation all three possibilities were investigated.

#### Variation 2: Altered outflow resistance

Following the sensitivity analysis on the $R_\text {out}$ parameter performed by Vinje et al. (2020), we ran two simulations where we either doubled (high resistance scenario) or halved (low resistance scenario) the outflow resistance $R_{DS}$ in Equation (10)

#### Variation 3: Changes to capillary filtration

Instead of modelling capillary filtration using the compartmental pressure difference between the capillaries and the capillary PVS, we set a constant capillary filtration rate. Vinje et al. (2020) decided on a filtration rate of of 0.16 ml/min in their simulations of constant capillary filtration, a rate we used as well.

#### Variation 4: Altered parenchymal CSF pathways

The exact magnitude and pathway of cerebral CSF flow is uncertain, due to both the small length scales of the cerebral microvasculature [55], and the possible large intrapersonal [60] and interpersonal [20] variations. These uncertainties allow for a certain degree of freedom when it comes to determining flow pathways and transfer coefficients. The simulations in this model variation investigated four scenarios with different relative magnitudes in the transfer coefficients. The changes to each transfer coefficient and their baseline values, with their corresponding case number, is shown in Table 4.

#### Variation 5: The importance of extracellular permeability 

There exist a great deal of uncertainty concerning the permeability of the extracellular space of the brain. Estimates range from $\kappa _{e}$ on the order of $10^{-17}$ m$^{2}$ [14] to $10^{-15}$ m$^{2}$ [17]. In this model variation, we aimed to investigate how a change in this parameter affect the flow of CSF and ISF, as well as if the response of the human brain to an infusion test changes with extracellular permeability. We have, in addition to the base model of $\kappa _{e} = 20$ nm$^{2}$ also tried a permeability of 10 and 100 times this value.

## Results

### The brain stabilizes quickly to equilibrium

**Fig. 4:**
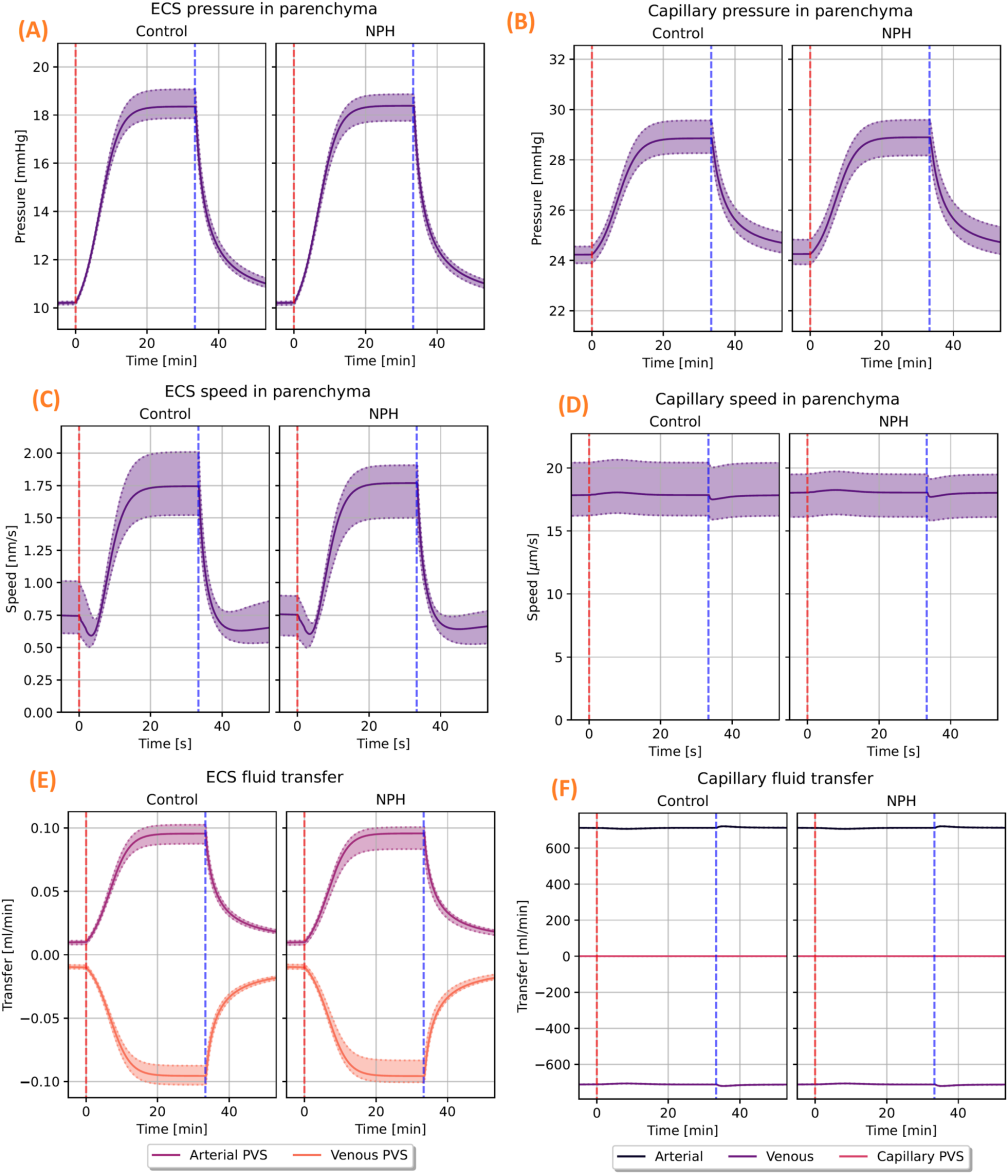
The computed pressure, fluid speed and fluid transfer rates in the ECS and cerebral capillaries. The shown pressure and fluid speed is volume averaged, while the fluid transfer panels show the integrated fluid transfer rate over the entire computational domain. Patient specific geometry and generic boundary conditions were used. On the top row, the computed volume averaged pressure in the ECS (**A**) and capillaries (**B**) is shown for both the control and iNPH groups. The middle row shows the corresponding average speed for the ISF in the ECS (**C**) and capillaries (**D**). Infusion starts right after $t=0$ min, marked by the red dashed line,and ends at $t=35$ min, marked by the vertical blue line. The panels at the bottom row show the net fluid exchange between either the ECS (**E**) or the capillaries (**F**) and their respective connected compartments. A positive value corresponds to a net inflow. These panels correspond to base model 1

The MPET model with subject specific geometries and generic boundary conditions (see Table 4) resulted in interstitial fluid superficial speed of 0.7 nm/s before the start of infusion in the control group and 0.8 nm/s in the iNPH group (Fig. 4C). Using a diffusivity $D= 1.3\cdot 10^{-10}$ m$^2$/s, corresponding to the MRI tracer gadubrutol ([19, 64]), and a length scale $L=0.1$ m (approximate brain length) this corresponds to Péclet numbers of 0.6. In both groups, the average fluid pressure in the extracellular space was 10.2 mmHg before the onset of infusion, and the fluid exchange rate between the ECS and both the arterial and venous PVS is less than 0.01 ml/min. The extracellular pressure and fluid speed is shown on the left in Fig. 4. Right after the start of the infusion, the ISF speed fell in all subjects, before quickly rising and stabilizing at an average speed of 1.8 nm/s (Pe = 1.4) in the iNPH group and 1.7 nm/s (Pe = 1.3) in the control group (Fig. 4C). The stablisation time was typically about 20 min from the onset of infusion, and can be seen on the left hand side in Fig. 4. Before the onset of infusion, CSF flow in the perivascular spaces was dominated by capillary filtration (data not shown), with a low CSF inflow rate in all subjects from the SAS to the arterial PVS. After infusion started, however, fluid transfer in the PVS was dominated by inflow from the SAS and ECS through-flow increased to 0.1 ml/min in both groups, as shown in (Fig. 4E). Blood flow remained almost constant throughout the entire simulation, shown in (Fig. 4F).

### Base model 1 - brain geometry does not explain increased ICP in iNPH during infusion

The difference in how the brain responded to an infusion test between the control and iNPH groups were found to be small when using generic boundary conditions. The total CSF inflow to the ECS from the arterial PVS was 0.1 ml/min in both groups, which is shown in (Fig. 4E). The largest observed difference in fluid speed between the groups was a 5.9% increase in the arterial PVS velocity in the iNPH group relative to the control group (data not shown). The relative difference in average pressure between the groups was bounded by 0.9 % which was reached in the venous fluid pressure.

### Base model 2 - Intracranial pressure differences differentiates iNPH patients from controls during infusion.

**Fig. 5:**
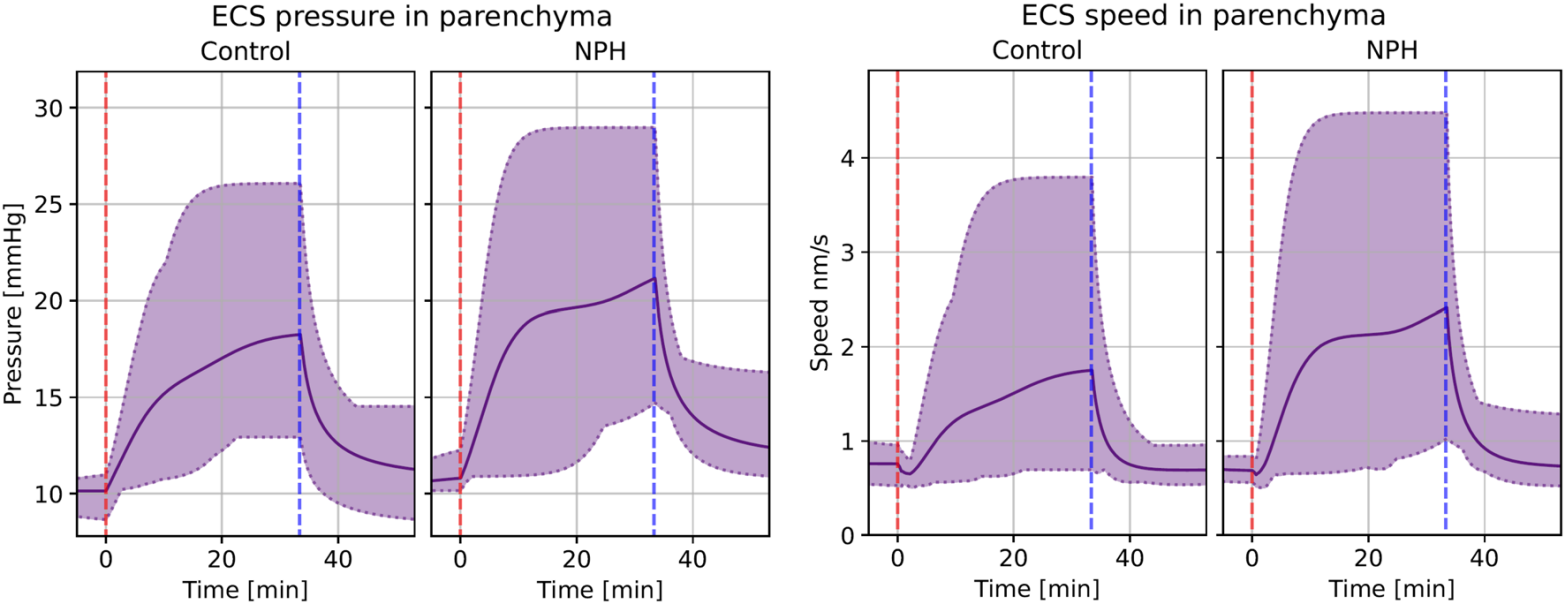
Volume averaged fluid pressure (left) and fluid speed (right) in the extracellular space for both the control and iNPH groups. The group average is shown as a solid line, while the dotted lines show the largest and smallest computed values for the pressure at any given time point. The red dashed line shows the start of infusion, while the blue dashed line marks the end of infusion. These curves show the simulation results when subject specific geometries and boundary conditions were enforced, and are from base model 2

The difference between the average iNPH patient and control increased after subject specific boundary conditions, rather than generic boundary conditions, were applied. The computed volume averaged fluid speed and pressure in the ECS for both groups is shown in Fig. 5. Within each of the CSF filled compartments, the average peak fluid pressure was higher in the iNPH group than in the control group during infusion. This corresponds to a pressure increase of 21–22% in the iNPH case. The venous blood pressure was 1.5 mmHg (12%) higher in the iNPH group at the end of infusion compared to the control group, while the arterial blood pressure was only 2% higher in the iNPH group.

The fluid speed within the perivascular compartments and the ECS was on average between 25% (in the capillary PVS) and 75% larger (in the arterial PVS) in the iNPH group than in the control group (data not shown). The peak ISF speed in the ECS was 2.4 nm/s (Pe = 1.8) for the iNPH group compared to 1.8 nm/s (Pe = 1.4) for the control group (Fig. 5). Throughout the entire infusion test, the relative difference between the two groups were always less than 56%. The relative difference in blood speed was lower than that of CSF speed, reaching at most 6.4% in the capillaries, with a slightly higher blood speed in the control group (data not shown).

**Fig. 6:**
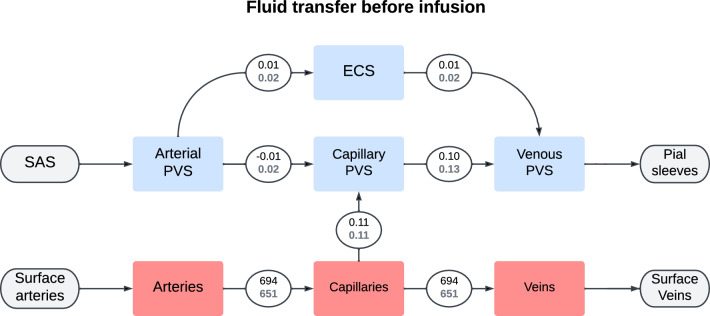
Flowchart showing the total inter-compartmental fluid flow right before the start of infusion ($t = -1$ min). The average fluid transfer rate in ml/min is shown in each connecting circle, with the upper black number being the average in the control group and the lower gray being the iNPH group. These numbers were computed using base model 2

**Fig. 7:**
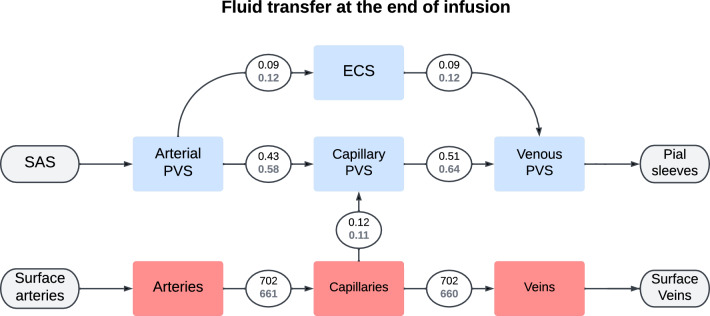
Flowchart showing the total inter-compartmental fluid flow at the end of infusion ($t = 50$ min). The average fluid transfer rate in ml/min is shown in each connecting circle, with the upper black number being the average in the control group and the lower gray being the iNPH group. These numbers were computed using base model 2

The total CSF flow through the brain was elevated at the end of infusion in the iNPH group. The average CSF flow from the SAS through the brain was 0.70 ml/min in the iNPH group and 0.53 ml/min in the control group, ie 28% and 38% of the combined volume of infusion and production flows through the glymphatic system of the healthy and iNPH patients, respectively. A fifth of this volume went through the ECS, reaching flow rates of 0.09 ml/min in the control group and 0.12 ml/min in the iNPH group at the end of infusion. The capillary filtration rate was nearly equal in the two groups at 0.12 ml/min and 0.11 ml/min in the control and iNPH groups respectively. A full overview over the flow patterns is shown in Fig. 7. Before the onset of infusion, CSF flow in the perivascular spaces was dominated by capillary filtration, shown in Fig. 6. In the control group, no CSF enters the arterial PVS at rest, while the total inflow rate for the iNPH group was 0.04 ml/min.

### Base model 3 - A generic brain geometry gives a reasonable approximation of mean flow in both ECS and PVS

**Fig. 8:**
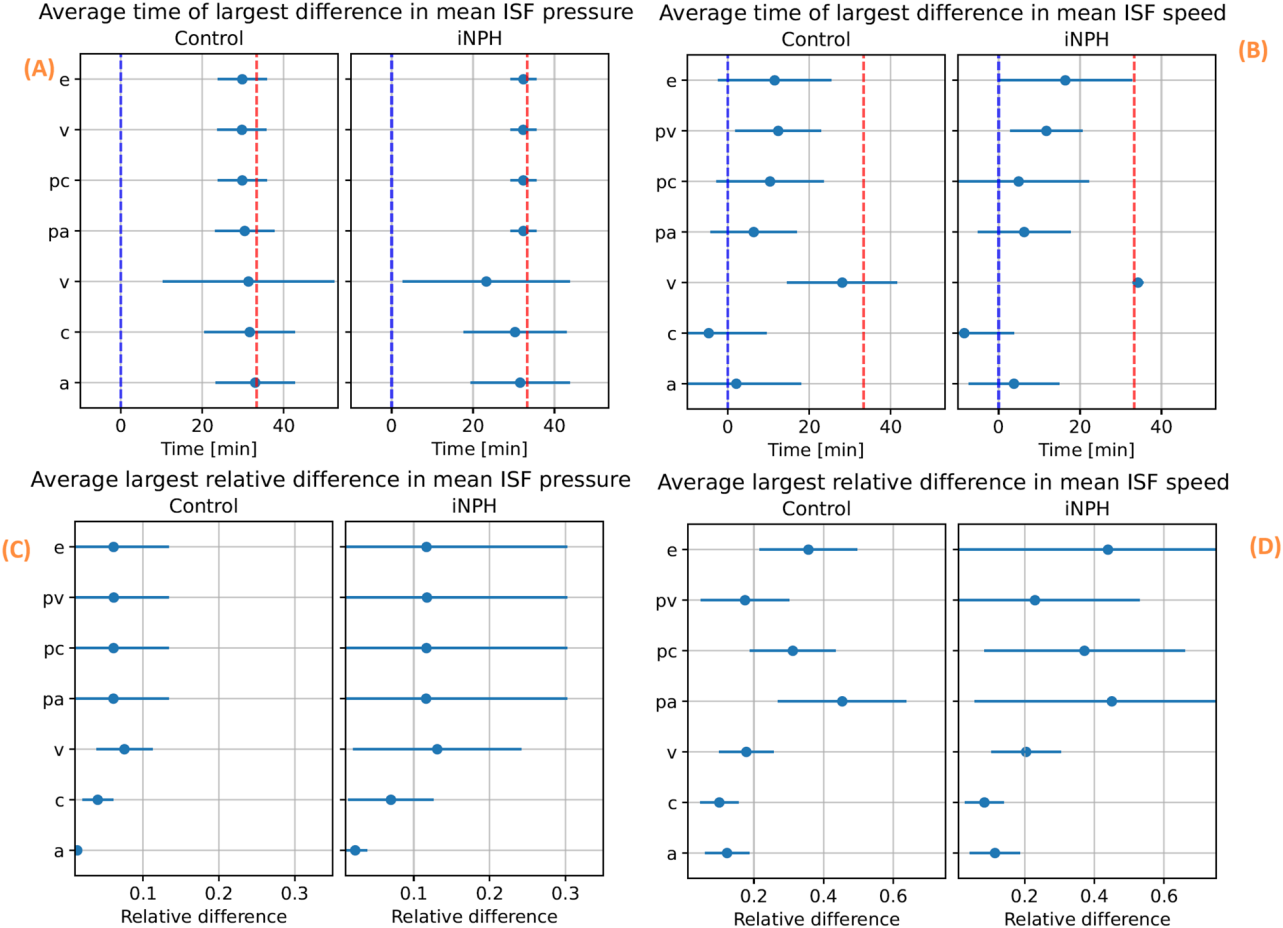
The relative difference between simulations performed on subject specific geometries and a generic geometry. For each subject, we ran a simulation using the subject specific boundary conditions on both the subject specific and group averaged geometries. Then, for each subject, we computed the relative difference in volume averaged fluid pressure (left) and fluid superficial speed (right) between the simulation result when using a subject specific geometry and an average geometry, and recorded at what time the maximal difference occurred. The top two panels, (**A**) and (**B**) show the average time point for the largest relative difference in pressure (**A**) and superficial speed (**B**) occurs. On the bottom row, the average largest relative difference achieved in terms of both pressure (**C**) and superficial speed (**D**) in each compartment and group is shown. In all panels, the compartmental group average is denoted with a blue dot, and the lines show the interval defined as the average ± one standard deviation. In panels (**A**) and (**B**) the start of infusion is marked by a blue dashed line, while the end of infusion is marked by a dashed red line. These results were computed using base model 3 and base model 2

For each subject, we performed one simulation with subject specific boundary conditions and subject specific geometry, and one simulation with subject specific boundary conditions and average geometry. For most subjects, the relative difference in pressure between the two simulations was largest close to the end of infusion for all compartments (Fig. 8A). Fluid speed in every compartment reached a maximal relative difference closer to the start of infusion (Fig. 8B).

The difference between subject specific geometries and average geometries in terms of mean pressure was, on average, slightly less than 10% different in the control group and slightly higher than 10% in the iNPH group. One notable exception, shown at the bottom of (Fig. 8C), was the arterial blood pressure, where the relative difference was less than 1% in both groups. Unlike the control group, there was a large intragroup variation among the iNPH patients in the PVS and ECS between the subject specific and average geometries. Here, the standard deviation was almost 0.2, and twice the size of the mean relative difference of 0.1.

In all compartments the fluid speed is more sensitive than the fluid pressure with respect to the brain geometry variations. Fluid speed is proportional to pressure gradient and the pial surface geometry is important in setting the average pressure gradient close to the pial surface. This is where the gradients and velocities are at their largest, as this is where the fluid enters and leaves the parenchyma. The largest relative difference in speed between a simulation with a subject specific geometry and average geometry was found in the arterial PVS. Here, the relative difference reached 40% with a standard deviation of ± 10% in the control group and ± 20% in the iNPH group (Fig. 8D). There was no discernible trend across the different compartments for when the speed difference is at its largest. In the arterial and capillary compartments the largest difference was reached before the start of infusion, while the time of greatest difference occurred at different times during infusion for the other compartments. The latest point of largest difference happened in the extracellular and venous compartments, close to 20 min after the onset of infusion.

### Changes in material parameters yield a changed cerebral waterscape

#### Variation 1 - Uncertainties in pericapillary channel width yields large variations in CSF flow

**Fig. 9:**
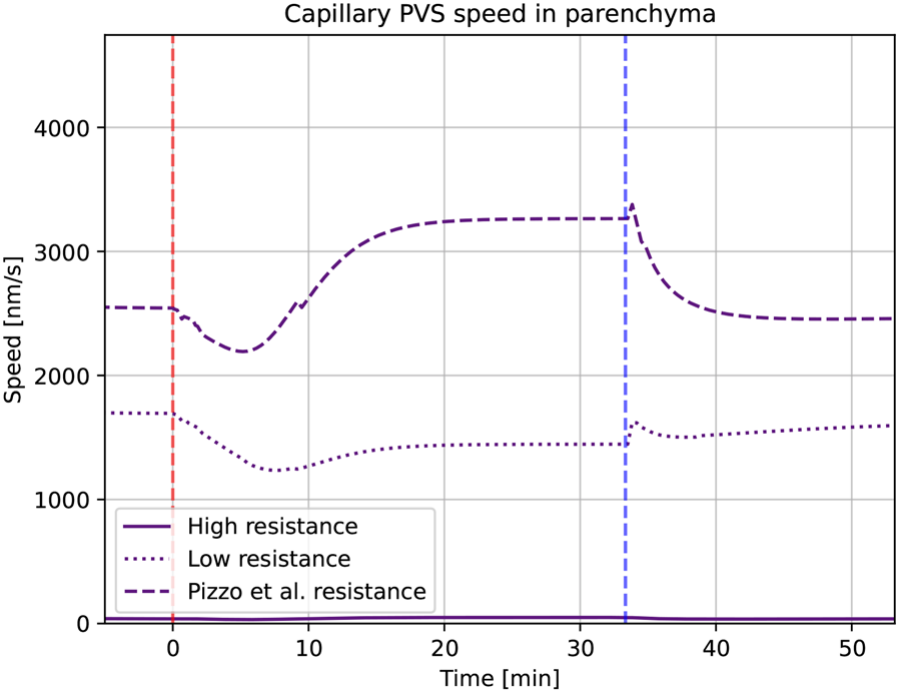
Average fluid speed in the entire capillary PVS as a function of time for each of the three different variations in pericapillary channel width. The perivascular permeability is a function of channel width, using equation (8) in [31] The red dashed line marks the start of, and the blue dashed line the end of infusion. Here “Low“ indicates the base model. These results are from model variation 1

**Fig. 10:**
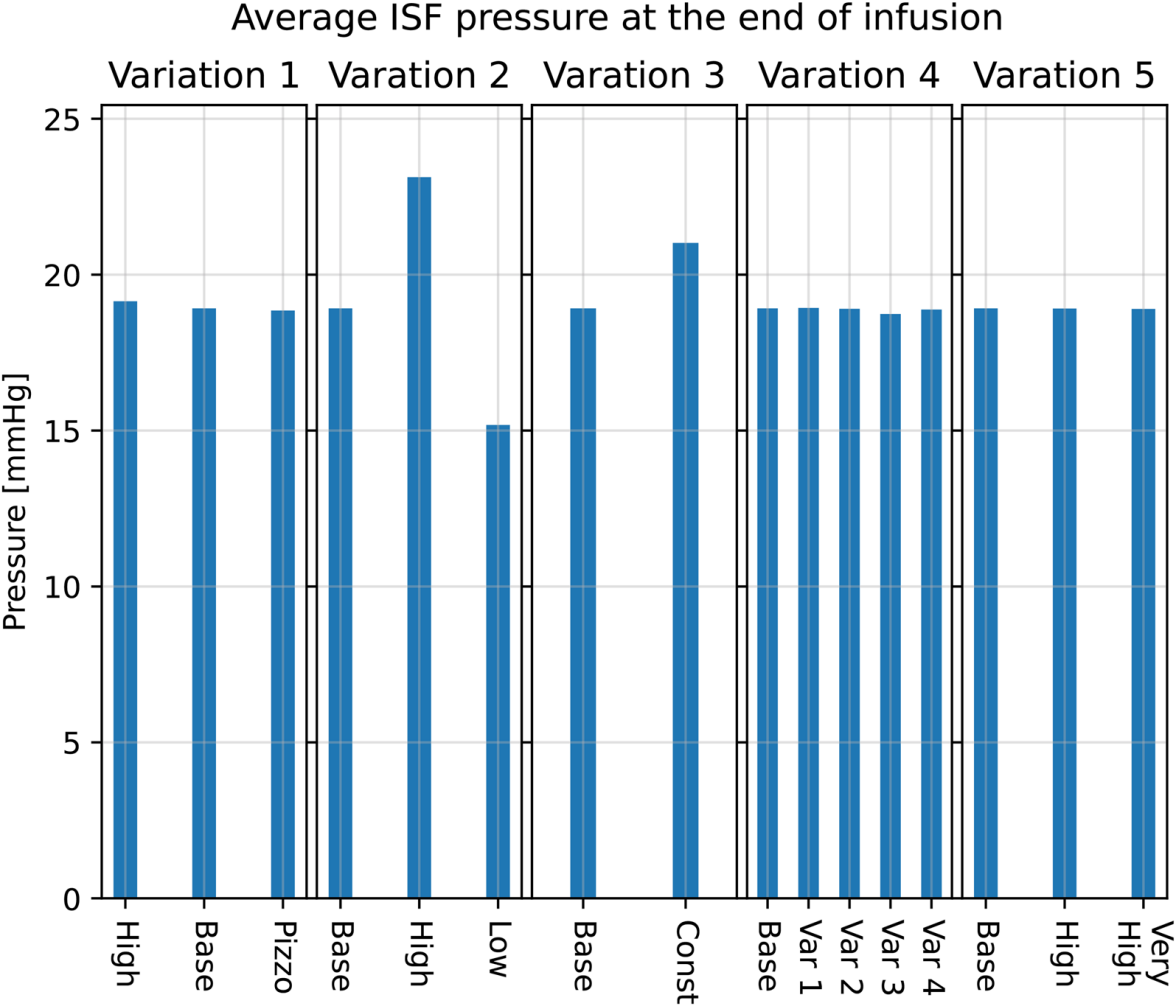
Different model variation effects on average parenchymal fluid pressure in the ECS at the end of infusion ($t =50$ min). In all panels, base refers to the base model explained in earlier sections. The scale is the same in all five bar plots. All simulations were performed on the average control group geometry with generic boundary conditions

The choice of pericapillary permeability, which itself is a function of the perivascular channel width following equation 8 from [31], had little effect on fluid pressure in the ECS, reaching an average of 19.1 mmHg in the high resistance scenario and 18.9 mmHg when using the resistance computed based on the image from Pizzo et al. [55]. With the base model, the ISF pressure reached 18.9 mmHg at the end of infusion. Figure 9 shows large variations in mean fluid speed. The pressure values are shown visually at the left side of Fig. 10. ISF speeds rose with increased flow resistance in the capillary PVS. In the high resistance scenario, fluid in the ECS reached an average speed of 3.7 nm/s, compared to 1.4 nm/s in the base model and 1.1 nm/s in the low resistance scenario (data not shown). The largest difference observed, however, occurs in the capillary PVS where the fluid plateau speed reached 3.9 nm/s in the high resistance scenario and 3300 nm/s in the lowest resistance scenario, which corresponds to the image taken by [55], is shown in Fig. 9.

#### Variation 2 - Changes in outflow resistance affect parenchymal CSF flow

A doubled resistance for CSF clearance through the arachnoid granulations resulted in an ISF pressure increase at the end of infusion of 4.2 mmHg on average within the parenchyma, from a plateau of 18.9 mmHg in the base model to 23.1 mmHg in the high resistance scenario. In the low resistance scenario, where the outflow resistance through the arachnoid villi was halved, the pressure plateau fell to 15.2 mmHg, shown in Fig. 10. Fluid speed in the ECS was similarly affected, with an increase of 0.6 nm/s to 2.0 nm/s (Pe = 1.5) in the high resistance scenario and decrease by 0.5 nm/s to a peak average speed of 0.9 nm/s (Pe = 0.7) in the low resistance scenario.

**Fig. 11:**
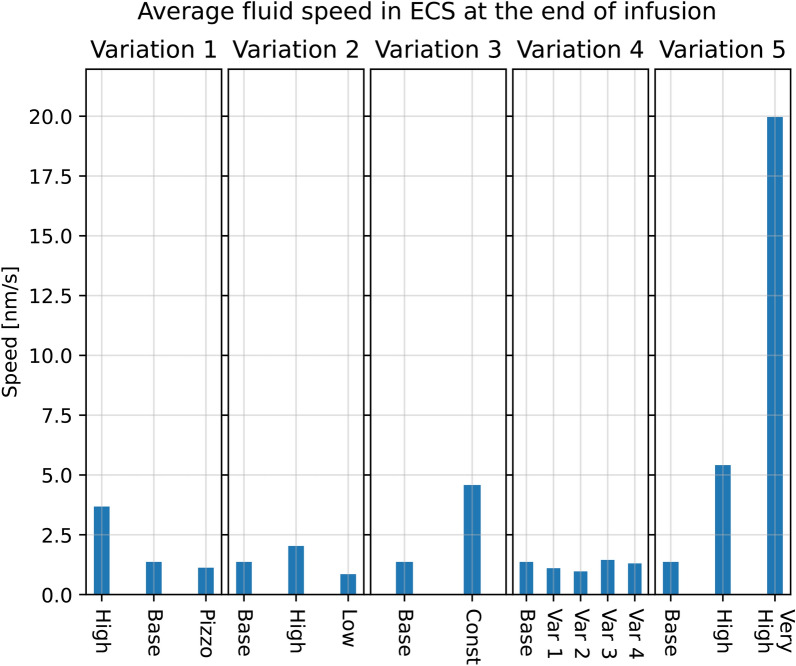
Different model variation effects on average parenchymal fluid speed in the ECS at the end of infusion ($t = 50$ min). In all figures, base refers to the base model explained in earlier sections. The scale is the same in all five bar plots. All simulations were performed on the average control group geometry with generic boundary conditions

#### Variation 3 - Constant capillary filtration yields higher flow rates

**Fig. 12:**
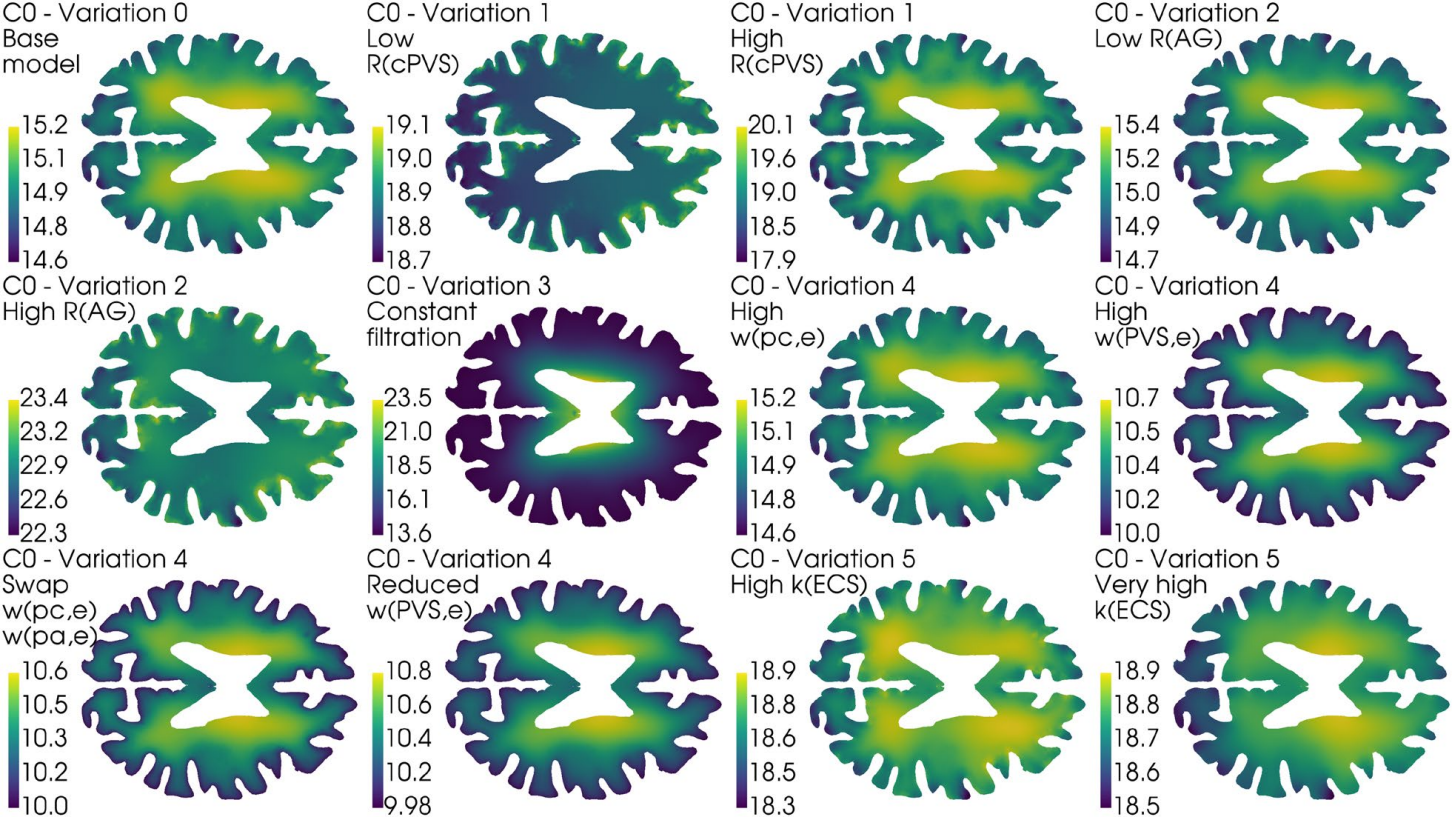
Axial slice of the average control brain geometry with the extracellular pressure field for each of the different model variations in Table 4. The base model is shown in the upper left, and serves as a point of comparison with every other result in this figure. The pressure field is computed at the end of infusion, and the pressure values in mmHg is shown in the colorbar to the left of each panel. All cases reveal pressure gradients within the parenchyma, and in the most extreme case (Variation 3, Constant filtration) the transmantle pressure difference is 9.9 mmHg. In most cases (except for Variation 1, Low $R_\text {cPVS}$ and Variation 2, High $R_{AG}$) the ISF pressure is largest close to the lateral ventricles. This ensures the ISF flow is mainly directed outwards from the ventricles towards the pial surface

A constant capillary filtration rate leads to an increase in fluid pressure and fluid speed in the ECS, shown in Fig. 10 and Fig. 11. The ISF pressure at the end of infusion increased from 18.9 mmHg in the base model to 21.0 mmHg, and the average fluid speed is increased from 1.4 nm/s to 4.6 nm/s (Pe = 3.5). While the pressure in the gray matter was lower here than in the base model, an elevated white matter pressure was found close to the lateral ventricles, shown in Fig. 12. These result show an increased transmantle pressure difference, defined here as the pressure difference between the ventricular walls and the cortex, going from 0.6 mmHg in the dynamic case to 9.9 mmHg for a constant capillary filtration.

#### Variation 4 - Changed transfer coefficients changed the total CSF flow through the brain

**Fig. 13:**
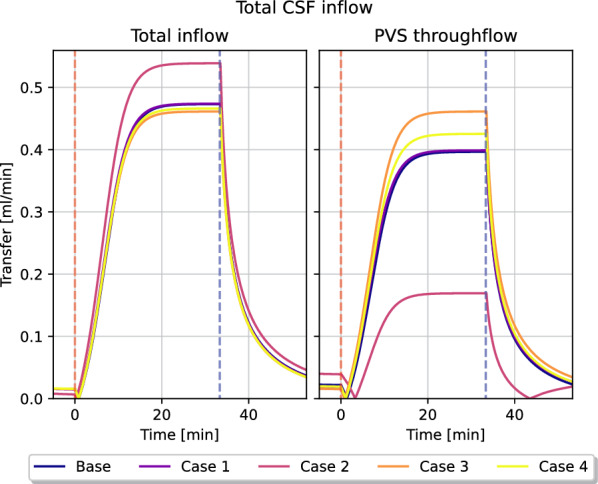
Total CSF inflow, and its chosen pathways for different variations in transfer coefficients. The figure to the left shows the total CSF volume flow rate that enters the brain through the arterial PVS, while the right figure shows how much of this fluid flows into the capillary PVS rather than the ECS. Infusion starts at $t=0$, and is marked by the red dashed line, and ends at the blue dashed line. These results are from model variation 4

Fluid transfer rate patterns between compartments remain similar in most of the transfer coefficient variations. In particular, we found a peak inflow rate of 0.45 ml/min into the arterial PVS in all but one variation. In these cases, fluid flow from the arterial PVS to the capillary PVS were around 0.40 ml/min and 0.45 ml/min, and flow from the arterial PVS to the ECS was limited. In one variation (case 2) the majority of CSF entered the ECS rather than the capillary PVS. The total inflow was 0.55 ml/min, of which less than 0.2 ml/min entered the capillary PVS (Fig. 13). The average fluid pressure is minimally affected by the changes in perivascular transfer coefficients, as can be seen in Fig. 10. The average fluid pressure in the ECS varied by at most 0.2 mmHg from the base model in the transfer coefficient variations. Fluid speed in the ECS remained almost unchanged as the transfer coefficients changed. The base model found a plateau speed of 1.4 nm/s (Pe = 1.1), with the different changes in transfer parameters giving a plateau speed of 1.4 nm/s (Pe = 1.1) at the highest and 1.0 nm/s (Pe = 0.8) at the lowest. The plateau speeds are shown second from the right in Fig. 11.

#### Variation 5 - Extracellular permeability changes do not affect parenchymal flow patterns

Changes in extracellular permeability $\kappa _{e}$ did not have a large impact on ISF pressure, where all permeability changes tested reached the same plateau pressure of 18.9 mmHg as the base model, shown in the middle graph of Fig. 10. The ISF speed, however, increased as the permeability rose, going from under 1.4 nm/s (Pe = 1.1) in the base model, to 2.0 nm/s (Pe = 1.5) in the high permeability scenario and 20.0 nm/s (Pe = 15) in the very high permeability scenario, shown in Fig. 11.

## Discussion

### Summary

 In our simulation model, when comparing the effect of 1) subject specific brain geometries, 2) subject specific intracranial pressures, and 3) variations in physiological parameters, we can conclude that the variations in physiological parameters cause the largest effects reaching up to a 14-fold difference between the lowest and highest computed fluid speed (Model variation 5). These parameters are also the most uncertain. Next, the variations caused by measured intracranial pressures are up to 75% (fluid speed) and 22% (fluid pressure), while the image based brain geometries cause variations of up to 6% (fluid speed) and 1% (fluid pressure). Finally, we revealed that for differences in ISF pressure to occur, outflow resistance from the SAS (here represented by the combined contribution of $R_\text {DS}$ and $R_\text {crib}$), or capillary filtration close to the ventricular wall may be of particular interest.

### Transport before infusion 

The base model, and most model variations, indicate that the transport is not convection-dominated. Before infusion, we found the average ISF superficial speed to be less than one nm/s , which yields Péclet numbers of 0.6 when using diffusion coefficient of Gadubrutol. These speeds are in the same order of magnitude as previously simulated superficial speeds [14, 43, 65]. The former of these two found that even with a pressure gradient of 1 mmHg/mm, they were not able to get interstitial fluid speeds greater than 1–10 nm/s (with our assumptions on diffusivity and length scale *L*, this correspond to Péclet numbers in the range of 0.8–8). In [43], our model was implemented, with some notable differences from the implementation in this article. Here, the authors reported a pre-infusion average speed of 2 nm/s (Pe = 1.5) in both groups, corresponding to a pressure gradient of 0.07 mmHg/mm. Our findings with the current model suggest a lower extracellular fluid speed than what was found in [43]. The only major change to the model implemented in [43] and this article is a reduction in pericapillary flow resistance and a inclusion of the $Q_{{\text {pvs}}}{(t)}$ in Equation (10). The difference in the magnitude of the ISF fluid velocity suggest a plausible link between cerebrospinal fluid dynamics in the PVS and the rate of flow in the ECS. Our simulations resulted in a pressure average 2.9 mmHg greater in the iNPH group than in the control group at the end of infusion. This pressure difference, indicating the iNPH group has a 22% higherpeak ISF pressure, is lower than the expected pressure difference in ICP between the two groups. The iNPH group has an outflow resistance $R_{out}$ which is 80% greater than the control group. This difference in pressure change between the measured CSF pressure and computed ECS pressure might be caused by the inclusion of the $Q_{{\text {pvs}}}{(t)}$ sink term in Equation (10). This term was included to account for the unphysically large CSF flow rates found in [43]. In [43], the authors, following the results derived by [31], excluded this term from the ODE modelling subarachnoid CSF pressure. When the full 3D model of the brain parenchyma was introduced, however, this assumption yielded flow rates of up to 3.0 ml/min through the PVS, more than twice the infusion rate. The $Q_{{\text {pvs}}}{(t)}$-term, while successful in bounding the perivascular flux, does also limit the pressure growth on the boundary. It is also worth noting that the pressure difference will, even in the absence of the $Q_{{\text {pvs}}}{(t)}$-term still not reach 80%, as the ISF pressure in the iNPH group in [43] is 60% greater than the control group. Hence, some of this difference between $R_{out}$ and plateau ISF pressure differences does evidently not stem from the sink term in Equation (10), but from other sources.

It can be tempting to suggest enforcing either the infusion pressure directly at the boundary, or to enable a backflow mechanism where (some of) the CSF leaving the venous PVS reenter the SAS, thus increasing the CSF pressure at the boundary. We believe neither of these approaches to be well-advised. The first approach would then inevitably end up in the same situation as [43], as this approach would lack a self-regulating mechanism for bounding the CSF flux through the brain. The second approach, which would necessarily lead to an increased fluid pressure in the SAS, is to our knowledge not quantified and would therefore be speculative, introducing more uncertainty into the model. A third possible approach is to tune the $\beta$-coefficients in the Robin boundary conditions, but the tests we performed on these parameters did not produce any noticeable effect on the plateau pressure. (Data not shown)

Earlier experimental work has suggested bulk flow velocities in brain tissue of around 0.2 $\mu$m/s [18, 66] (corresponding to Pe = 150). In the ECS, ISF superficial speeds in the range of 0.58 to 2.50 $\mu$m/s (Pe = 446 to Pe = 1900) have been reported (see the computational study by [17]). These fluid speeds are primarily driven by both a larger pressure gradient than what we found between the arterial and venous PVS, and the assumption of a larger extracellular permeability than the one we used in our model. In a mouse model, [67] found average ISF velocities on the order of 3–10 nm/s (Pe = 2.3 to 7.7). Velocities of this magnitude are too small for convection to be the dominating cause of transport of solutes. In our study, we have investigated the effect of changes in extracellular permeability as seen in the middle of Fig. 11. Even with an increase of extracellular permeability of two orders of magnitude (“Very high“ case), the average ISF speed is found to be only 20 nm/s (Pe = 15) at the end of infusion. Therefore, it is unlikely that the large difference in ISF speed can be explained by the difference in permeability.

In [17] they manually set a pressure difference between the arterial and venous PVS, and an extracellular permeability. For each set of conditions, they looked at the induced flow in the ECS. To get speeds in the range of 0.58 to 2.50 $\mu$m/s, [17] had to set a pressure of 0.8 mmHg between the arterial and venous PVS. With their chosen distance of 250 $\mu$m between the arterial and venous PVS, this pressure difference would lead to a pressure gradient of 3.2 mmHg/mm, significantly larger than pressure gradients in our model. This gradient is in the same magnitude as what [48, 68] found to be necessary to find realistic CSF inflow to the penetrating PVS in mice, at 1.2–3.3 mmHg. Yet, as [48, 68] points out, this pressure change is unlikely as the largest possible transmantle pressure difference in humans is believed to be less than 1 mmHg, see [69]. This limit is further corroborated by [30], whose viscoelastic model predicted the necessary transmantle pressure difference for the formation of ventriculomegaly to be 1.76  mmHg. Hence, we find it unlikely for there to be sustained pressure gradients of this magnitude in healthy adults. Our computed average speeds in the ECS correspond to pressure gradients of 0.03 mmHg/mm, and [43] found an average pressure gradient of 0.07  mmHg/mm. Even if the pressure gradients in our study appear small, these gradients are slightly higher than pulsatile pressure gradients measured experimentally at around 0.0015 mmHg/mm [70]. Static pressure gradients over the cerebral aqueduct needed to transport a production of 0.5 L CSF per day has been estimated even lower, at $10^{-5}$ mmHg/mm [70].

### The sensitivity of the brain to infusion tests 

The response of the brain to an infusion test has been modelled by several authors, and some articles has also used multi-compartment models. The model by [31] served as a baseline for many of our parameter choices, and in many aspects our model agrees with the results of [31]. In [31, Fig. 3], the authors show that the intracranial pressure stabilizes 20–30 min after the onset of infusion, which is in agreement with in vivo infusion tests [2]. Unlike [31], we find the amount of CSF entering the brain to increase during an infusion, rather than decrease. This difference might stem from the 0D nature of the model in [31], where constant capillary filtration was applied everywhere in the brain. In the present study, we assumed capillary filtration only at the ventricular surface. Regardless of model choice, we find that about a third of the infused CSF enters the brain from the SAS, as shown in Fig. 13. We remark that it was reported in [19] that up to a third of the intrathecal contrast entered the brain.

Poroelastic models has also been used to investigate infusion tests. In [71], the authors used a two-compartment poroelastic model to describe the spatial propagation of a sudden increase in ICP in terms of strain and displacement of brain tissue. Notably, they found that even though CSF pressure remained virtually constant throughout the parenchyma, both displacement and strain fell rapidly from the pial surface and into the parenchyma. In most of our model variations, the largest pressure gradients, and hence also fluid velocities are found in the gray matter. This is shown in Fig. 12, and also documented in [43, chp. 7].

The tracer infusion experiments of [7, 72] and [54] can be regarded as an almost-infusion like scenario due to the relative size between murine and human brains and their infusion rates [31]. While direct numerical comparisons might be unfounded, we believe a qualitative comparison might be appropriate. We note, for example that [72] found a large extracellular bulk flow at over 1 $\mu$m/s in the cerebral extracellular space of mice after injecting tracer directly to the interstitium. Hence, it is clear that bulk flow might be possible if the local pressure gradient is large enough, as might happen during infusion. In alignment with [54] and [7], we find the perivascular fluid speed to be significantly higher than interstitial fluid speed in all stages of our simulation. [32] used MPET to investigate infusion tests and found that the pressure in the PVS and ECS, which in their article was a single combined compartment, should follow the intracranial pressure (pressure in the SAS) during infusion. This finding is supported by our own results, as the pressure graphs in e.g., Fig. 4 follow the ICP in [31], which we used as the model for CSF pressure in the SAS.

The computed pressure fields shown in Fig. 4 predict that there is little to no difference between healthy individuals and iNPH patients in response to infusion, given that the geometry is the only difference between the groups. In that case, the largest difference between the control and iNPH group, as a group average, is a 5.9% larger fluid superficial speed in the venous PVS. However, in our experimental data, the average $R_{out}$ in the iNPH group was 18.1 mmHg/(ml/min) compared to 10.0 mmHg/(ml/min) in the control group. With an infusion rate of 1.5 ml/min, this would correspond to a difference of 12.5 mmHg between the two if we assume the response to be linear. The assumption of linearity is unlikely to be true, as the linear relationship between pressure and infusion rate vanish if ICP surpass 26 mmHg [73]. Yet, a difference of 6% which is only found in a single quantity of interest within a single compartment, seems too small to be an accurate representation of the groups. Therefore, we find it likely that other parameters than the geometry differ between the groups.

The difference in response between the iNPH and control group grew when subject specific boundary conditions based on the infusion test were implemented. Now, we observe a 21–22% difference in average fluid pressure and between 25% and 75% in fluid speed in all CSF-filled compartments. The difference in ISF pressure and speed is shown in Fig. 5. The boundary conditions represent the CSF dynamics happening at the surface of our computational domain, ie. the brain surface. However, it is not clear that the lumped parameters of the infusion test can be translated to uniformly distributed boundary conditions in such a simple way as modelled here. In particular, the increased resistance may also express increased resistance of some of the glymphatic pathways within the parenchyma. In particular, our model predicts a positive correlation between $R_{out}$ and all of our quantities of interest. Figure 5 shows the difference between the control and iNPH groups in terms of ISF pressure and superficial speed, and the only difference between the groups is the boundary conditions. Here, both the pressure and fluid speed is higher on average within the iNPH group. In contrast, [22] discovered that the clearance of CSF tracer from the brain after intrathecal injection was significantly delayed in iNPH patients compared to healthy adults. Furthermore, CSF dynamics and transport in the ventricles differ substantially between the two groups [21, 22]. These observations may suggest that our assumption of equal permeability in all compartments in both groups might be incorrect.

While the boundary conditions seem to be important for differentiating between an iNPH patient and a healthy individual, the case for using subject specific geometries is more complicated. The largest relative difference in pressure between an average control and iNPH geometry and a subject specific geometry is found to be around 5% and 10% respectively, as shown in (Fig. 8C). However, the difference in speed increases to over 20 – 40% in the PVS and ECS in both groups (Fig. 8D). A possible explanation lies in the fact that the averaging process yielded a smoother and smaller cortical surface. The largest pressure gradients in our model occur in the gray matter, see either Fig. 12 or [43, chp. 7], and a reduced cortical area from the smoothing process is therefore a likely candidate for the difference in velocity between average and subject specific geometry. The decreased cortical surface area and relative difference between gray and white matter would yield reduced average pressure and velocity. It is also worth noting that the largest difference in average pressure, shown in (Fig. 8A), happens at the end of infusion, while the largest difference in fluid speed occurs close to the middle of infusion in all compartments. Hence, as the difference in pressure between subject specific and average geometries is largest at the end of the simulation, we find it likely that this difference show the effect of subject specific geometries on the pressure plateau part of the infusion. As ICP plateau pressure is used to compute $R_{out}$, it seems therefore unlikely that a subject specific geometry is likely to yield a different result in terms of one of the most common indicators measured during infusion. The CSF superficial speed, however, differs between the two geometries at almost the beginning of infusion, indicating that there might be interesting flow patterns that are geometry induced which are not a function of the plateau pressure.

Our model included the possibility of flow in capillary PVS. This flow has not been documented experimentally. However, with the approximate size of PVS as shown by Pizzo et al. [55], the capillary PVS resistance is very low, resulting in capillary PVS velocities of around 2–3 $\mu$m/s. With all capillary PVS resistances tested in this work, most CSF/ISF flows via capillary PVS rather than the ECS [43]. Particles in these spaces would thus move around 15–20 cm per day, providing a strong clearance mechanism, even without bulk flow in tissue, considering that brain wide clearance occurs on the day scale [23]. If bulk flow occurs only in PVS, diffusion as a transport mechanism is more than sufficient over the small distances from ECS to PVS (25–50 $\mu$m) over such a time frame [74]. However, if velocities in capillary PVS are as high as reported when using the low resistance obtained with data from Pizzo et al. [55], resistances in the arterial and venous PVS might be expected to be lower as well, implying possibly faster clearance rates. We also note that our model found a small drop in average fluid speed in the ECS at the start of infusion. From our results, we find the most likely explanation for this phenomenon to be the change of dominant CSF source from capillary filtration in the capillary PVS, to infused CSF from the ECS. This change in source also reconfigures the velocity field, as the CSF from capillary filtration is introduced over the entire parenchyma, while CSF from the SAS only enters through the pial surfaces.

We set a very low transfer coefficient between capillary PVS and the ECS. Hence, our model yields two possible CSF flow pathways, either flowing through the PVS, or flowing from the arterial PVS to the ECS before reentering the PVS alongside the veins. In most permutations of transfer coefficients in variation 4, the majority of CSF entering the brain through the arterial PVS went to the capillary PVS at the end of infusion, shown in Fig. 7 and Fig. 13. The one notable exception, shown in magenta in Fig. 13 (case 2), is the one where the transfer coefficients between the perivascular compartments and the ECS was increased with a factor 10 (arterial PVS and venous PVS) and a 100 (capillary PVS). This indicates a clear preference for perivascular bulk flow, as suggested by [13], rather than the convective flow in the ECS as proposed by, e.g., [7, 17].

When the capillary filtration was applied as a boundary condition at the ventricular wall, rather than a non-zero fluid transfer rate term between the capillaries and their perivascular spaces, we found significant changes to the cerebral waterscape. The computed transmantle pressure difference with a constant capillary filtration is found to be 10 mmHg, shown in Fig. 12. These values are unlikely, as a transmantle pressure gradient of this magnitude is significantly larger than the proposed limit of 1.76 mmHg required to form ventriculomegaly [30]. While this transmantle pressure gradient is found in the infusion setting, a transmantle pressure gradient this large could very well lead to significant damage to brain tissue, something not observed in subjects who have undergone infusion testing.

## Limitations and further work

Our study is to our knowledge the first to model infusion test in a subject specific manner, in the context of the glymphatic system. In order to arrive at such a model we have made a number of simplifications. We considered the CSF dynamics in the subarachnoid space and possible spatial variations in in- and outflow routes to be of minor importance during infusion and that the pressure increases synchronously in the whole subarachnoid space. Furthermore, we have disregarded the spatial deformations and used a scalar valued permeability.

As in [31], the majority of the fluid leaves the system along other pathways than the glymphatic pathway (0% at rest and 28% during infusion in the healthy and 12% at rest and 38% during infusion in the iNPH patients, respectively) and the resistance of the pathway is only a fraction of lumped CSF resistance as described by Davson’s equation, cf [73] where resistance of 22.3 mmHg/(ml/min) was established in a group of thirty iNPH patients. We remark that in [19] it was observed that about 1/4 of the CSF tracer (gadobutrol) administered by intrathecal injection entered the brain after about 6 h. To what extent these findings are in conflict or not is not clear and cannot be revealed by the model in this paper as tracer concentrations are not part of the predictions. Furthermore, we note that it has been observed that the cross-sectional area ratio between vasculature and PVS differ between the arterial and venous cites. In  [75] it was shown that venous ratio is only 0.13. We also remark that the $\beta$-values used for the Robin boundary conditions in Table 3 are determined in order to have a response time-scale around 30 min. We remark that the values are orders of magnitude higher than corresponding values determined at the microscale for the blood-brain barrier or the endfeet sheet [76]. In our study here we have used lumbar pressure measurements directly as boundary conditions within the cranium. There are small pressure gradients within the CSF that may have then been ignored. For example [70, 77] suggest pressure gradients of the order of a few mmHg per meter. A further limitation of the model is in the reliance on murine observations in determining the model parameters. We have shown that material parameters are important, with some model variations changing ECS fluid speeds by an order of magnitude (see Fig. 11). Hence, any systematic difference in these material parameters between mice and humans will yield significant and systematic errors in our model predictions.

Finally, we mention that based on the modeling paper [67], we mostly excluded the connection between ECS and capillary PVS in our models. To the authors’ knowledge, the parameter of this pathway is not available in the current literature.

## Conclusion

 Infusion tests measure the resistance of the CSF efflux routes and are well established for assessment of iNPH patients. The relationship between this test and the glymphatic system has so far not been adequately modelled or explained. Here, we introduced a subject specific seven compartment model, involving both the vascular, perivascular and extracellular compartments and their interactions under infusion. Surprisingly, we found that the subject specific geometries only play a minor role as compared to the CSF pressure increase under infusion and that other model parameters are more important, such as the infusion pressure, the permeabilities and the transfer coefficients. A considerable amount, but not the majority, of the infused CSF passes through the glymphatic system, according to our computations.

## Data Availability

No original datasets were used in the present study. All parameters used in the model are described in the methods section. The numerical code and the average control brain geometry is freely available at https://github.com/larswd/MPET-model-iNPH.
